# Objective physical activity and Alzheimer's disease burden in the population‐based Rotterdam Study

**DOI:** 10.1002/alz.70655

**Published:** 2025-09-18

**Authors:** Phuong Thuy Nguyen Ho, Meike W. Vernooij, Trudy Voortman, María Rodriguez‐Ayllon, Julia Neitzel

**Affiliations:** ^1^ Department of Radiology and Nuclear Medicine Erasmus MC University Medical Centre Rotterdam the Netherlands; ^2^ Department of Epidemiology Erasmus MC University Medical Centre Rotterdam the Netherlands; ^3^ Biomedical Research Institute of Malaga (IBIMA Platform Bionand) Malaga Spain; ^4^ Prevention and Health Promotion Research Network (redIAPP) & Chronicity Primary Care and Health Promotion Research Network Madrid Spain; ^5^ Department of Epidemiology Harvard T.H Chan School of Public Health Boston Massachusetts USA

**Keywords:** Alzheimer's disease, amyloid‐beta, dementia, physical activity, plasma biomarkers, population‐based

## Abstract

**INTRODUCTION:**

Physical activity is linked to lower dementia risk, but its connection to Alzheimer's disease (AD) pathology remains uncertain. This study examined the relationship between objectively measured physical activity and early AD biomarkers in cognitively unimpaired adults.

**METHODS:**

Accelerometer‐measured physical activity and plasma AD biomarkers (beta‐amyloid [Aβ]42/Aβ40, p‐tau217) were assessed in 242 participants (age = 63.37 [54–79] years) of the population‐based Rotterdam Study. Cortical Aβ was assessed via ^18^F‐florbetaben positron emission tomography (PET) 7 years later. Robust regression assessed the relationship between physical activity, plasma AD biomarkers, and Aβ PET burden, while compositional analysis examined how the time‐use composition relates to AD outcomes.

**RESULTS:**

Physical activity was not associated with plasma Aβ42/Aβ40, p‐tau217, or brain Aβ burden 7 years later. Reallocating awake sedentary time to physical activity showed no association with AD biomarkers.

**DISCUSSION:**

No relationship was identified between physical activity and AD biomarkers, suggesting physical activity might affect dementia risk through other pathways or in an earlier life phase.

**Highlights:**

- Physical activity was not associated with plasma beta‐amyloid (Aβ)42/Aβ40 nor p‐tau217.
- No association was observed between physical activity and brain Aβ 7 years later.
- Age, sex, and apolipoprotein E ε4 carriership did not moderate these relationships.
- Reallocating 30 minutes of sedentary time to physical activity resulted in no change in Alzheimer's disease (AD) biomarkers.

## 1 BACKGROUND

Dementia is often regarded as having a multifactorial origin, with both biological and lifestyle factors contributing to its development.^1^ The Lancet Commission identified physical activity as one of the modifiable factors that, in combination, could reduce up to 45% of dementia cases.^2^ Studies have shown a long‐term link between physical activity and reduced risk of dementia, including Alzheimer's disease (AD).^3^, ^4^ This relationship seems bidirectional: physical activity can decrease the risk of developing AD, whereas clinical AD symptoms can hinder physical activity.^5^ Although AD impairs physical activity through mobility limitations, the mechanisms by which physical activity may influence AD remain unclear. One hypothesis is that physical activity reduces the production or promotes the removal of early AD pathology.^3^ To investigate this, the current study examined the relationship between physical activity and early AD biomarkers.

Aggregation of beta‐amyloid (Aβ) peptides into extracellular plaques and hyperphosphorylated tau proteins into fibrillary tau tangles are key hallmarks of AD.^6^, ^7^ Early AD pathology is characterized by a decreasing ratio of long to short Aβ oligomers (Aβ42/Aβ40), reflecting the selective depletion of Aβ42 from blood due to growing brain deposition.^8^, ^9^, ^10^ Abnormal Aβ levels become detectable on positron emission tomography (PET) scans through increased uptake of a radioactive tracer that specifically binds to cortical Aβ plaques. Elevated levels of phosphorylated mid‐region tau (p‐tau), particularly at codon 217 (p‐tau217), in blood rise in response to Aβ deposition and reflect Aβ‐mediated tau phosphorylation.^8^, ^11^ Thus, plasma Aβ42/Aβ40, p‐tau217, and Aβ PET mark the initial stages of AD pathology, often occurring decades before the onset of clinical symptoms.^12^, ^13^

We have searched the literature on physical activity and AD biomarkers and identified three key gaps. First, despite the promise of AD plasma biomarkers to monitor the effects of lifestyle factors, few studies have investigated their relationship with physical activity, and current evidence remains inconclusive.^14^, ^15^, ^16^, ^17^ Most studies investigated plasma Aβ42/Aβ40 in participants with mild cognitive impairment (MCI) and early symptomatic AD, reporting no association.^15^ A recent study linked higher physical activity to lower plasma p‐tau217 levels in a sample primarily composed of individuals with cognitive impairment.^18^ Importantly, most studies relied on self‐reported activity,^15^ which is prone to recall bias. Objective measures like accelerometry offer a more reliable alternative by continuously capturing movement across intensity levels. However, their relationship with plasma p‐tau217 levels in cognitively unimpaired individuals remains unexplored.

Second, few studies have examined the link between objective physical activity measures and amyloid PET burden.^14^, ^19^, ^20^, ^21^ Marino et al. (2024) found no difference in activity pattern by Aβ status in 109 participants of the Baltimore Longitudinal Study of Aging using accelerometry.^20^ Kimura et al. (2020) found no association between walking steps collected from wristband sensors and brain Aβ in 855 Japanese adults with MCI.^19^ Yet, MCI reflects an advanced disease stage, potentially obscuring the relationship between physical activity and Aβ PET in earlier stages.

Third, time‐use data are inherently compositional—representing parts of a finite whole (24 h/day).^22^, ^23^, ^24^ Because an increase in one behavior reduces time spent in another, traditional statistical methods treating physical activity as an independent variable can produce biased inferences. Compositional data analysis appropriately accounts for this co‐dependence. Prior studies have shown that spending more time being physically active relative to sedentary time is related to better cognitive performance and improved cardiometabolic health.^23^, ^24^, ^25^, ^26^ No study has performed a compositional analysis incorporating AD biomarkers.

**RESEARCH IN CONTEXT**

1. **Systematic review**: Studies identified in a PubMed search showed inconsistent associations between physical activity and the accumulation of Alzheimer's disease (AD) biomarkers. Existing literature largely relies on self‐reported activity data from convenience or selective samples, underscoring the need for population‐based studies with objective measures of physical activity. Few studies have combined plasma and imaging AD biomarkers to assess the associations between physical activity and AD pathology; notably, compositional analysis remains unexplored in this context.
2. **Interpretation**: Our study found no association between concurrent physical activity and plasma AD biomarkers (beta‐amyloid (Aβ)42/Aβ40 and p‐tau217), or between baseline physical activity and cortical Aβ burden on PET imaging 7 years later. Age, sex, and apolipoprotein E ε4 carriership did not moderate these relationships. Reallocating time from sedentary behavior to physical activity was not linked to changes in AD biomarker levels.
3. **Future directions**: Future research should explore additional/other aspects of physical activity (e.g., activity patterns, in combination with specific cognitive tasks) and examine the role of physical activity in early to mid‐adulthood, when AD pathology begins to accumulate.

This study investigated the relationship between objectively measured physical activity and AD biomarkers in cognitively unimpaired adults. At baseline, we measured accelerometer‐based physical activity and plasma biomarkers (Aβ42/Aβ40, p‐tau217) in 242 participants from the Rotterdam study (RS). Seven years later, participants underwent Aβ PET imaging. To fill the identified research gaps, we focused on (1) cross‐sectional associations between physical activity and plasma AD biomarkers; (2) associations between baseline physical activity and Aβ PET accumulation at follow‐up; and (3) a hypothetical scenario in which awake sedentary time is replaced by physical activity to examine its potential impact on AD biomarkers using compositional analysis.

## 2 METHODS

This study was embedded in the prospective population‐based RS. Participants were recruited from the district of Ommoord, Rotterdam, the Netherlands.^27^, ^28^, ^29^ The RS includes four subcohorts: RS‐I, which began in 1990 with 7983 participants aged 55 or older, was followed by RS‐II in 2000 with 3011 new participants aged 55 years old or older.^30^ In 2006, RS‐III began with 3932 participants aged 45 or older.^28^ RS‐IV commenced in 2016 with 3005 participants aged 40 or older. Approximately every 3 to 6 years, participants undergo a home interview and are invited to a dedicated research center for extensive examinations. Since 2005, participants have also undergone brain magnetic resonance imaging (MRI) examinations following a standard imaging protocol.^27^ Study details were published previously.^28^ All participants provided written informed consent. The RS was approved by the Medical Ethics Committee of Erasmus Medical Center.

From 2011 to 2016, a subsample of 2778 participants was randomly invited to wear an accelerometer for 1 week, with 2338 participants agreeing to participate (response rate 84%).^31^ Accelerometry data were considered valid if they met the following criteria: (1) at least 4 days of recording with at least 1200 minutes/day (*n* = 192); and (2) no technical errors (*n* = 122). In the end, 2024 participants had valid accelerometry data.

From 2018 to 2021, 18F‐Florbetaben PET imaging was performed in a subsample of the RS‐II and RS‐III cohorts. Participants were invited to the PET study if they met the following criteria: (1) at least 60 years old; (2) had a brain MRI acquired between 2011 and 2016, and brain segmentation yielded valid results based on visual inspection; (3) had no PET contraindications; and (4) did not have a diagnosis of dementia.^32^, ^33^ More details of the PET study design were previously described.^32^ Out of 2068 eligible participants, 1697 were invited, and 645 participants agreed to undergo PET imaging (response rate 38%). In total, 639 PET scans were acquired. Plasma biomarkers were also collected from this subsample between 2012 and 2015.

**FIGURE 1:**
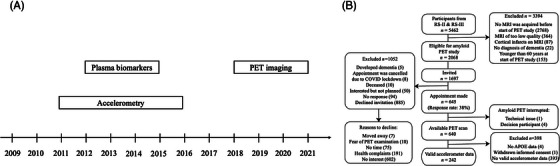
(A) Study design and (B) participant selection.

Participants were included in the analyses if they had a valid PET scan, APOE4 data, and accelerometry data, yielding a total sample of 242. A flowchart of the study design and participants’ selection is presented in Figure 1. To evaluate potential selection bias, we compared the characteristics of participants eligible for PET imaging with those included in this study (Table S1). The current sample included more men, was younger, and was more highly educated.

### 2.1 Physical activity

Participants were asked to wear a triaxial accelerometer (GeneActiv; Activinsights Ltd, Kimbolton, Cambridgeshire, UK) on their non‐dominant wrist for 7 consecutive days and nights, removing it only for bathing. They also kept a sleep diary during this period. More details on the processing of the accelerometry data were described by Hofman et al. (2022).^34^ Briefly, we used the PAMPRO software and Python (V.2.6.6) to process the data, excluding non‐wear time from the analyses.^34^, ^35^, ^36^ Average acceleration throughout the day, measured in milligravitational units (mg), reflects daily physical activity levels. Changes in accelerations over time correspond to both movement and the intensity of that movement; thus, higher acceleration indicates more vigorous physical activity.

Then, we categorized activity based on a validated acceleration threshold: sedentary time (< 48 mg), light (48 < 154 mg) (LPA), moderate (154 < 389 mg), and vigorous (> 389 mg) physical activity.^35^ For our analysis, moderate and vigorous physical activity were combined into moderate–vigorous physical activity (MVPA) and LPA and MVPA were combined into total physical activity (minutes/day).^34^ Sleep duration was computed using a validated algorithm that combined information from the accelerometry and sleep diary.^37^ To compute awake sedentary time, we subtracted sleep duration from total sedentary time. The 24‐hour day was divided into LPA (minutes/day), MVPA (minutes/day), awake sedentary time (minutes/day), and sleep time (minutes/day).

### 2.2 Amyloid PET

PET scans were acquired approximately 90 to 110 minutes after intravenous injection of 300MBq ^18^F‐florbetaben (Neuraceq, Life Molecular Imaging GmbH) for 20 minutes in listmode on a Siemens Biograph mCT PET/computed tomography (CT) (Siemens Healthineers). The data were processed according to an established pipeline.^32^, ^33^ Briefly, we reconstructed listmode data into four frames of 5 minutes and one frame of 20 minutes on a 400 × 400 matrix and corrected all images using a low‐dose CT scan (120 kVp, 40 quality reference mAs) acquired in advance. Next, we co‐registered participants’ CT scans with their T1‐weighted structural images using rigid body registration (SPM v12) and applied these transformations to align participants’ PET images. The high‐resolution T1 images were also used to derive anatomical parcellations from FreeSurfer (v5.1.0), which was later applied to the PET images to define regions of interest. A combination of frontal, cingulate, lateral parietal, and lateral temporal regions and a cerebellar reference region was utilized to calculate the average cortical standard uptake value ratio (SUVR). The SUVR values were the primary outcome of interest in this study. Aβ positivity was defined using an algorithm combining SUVR and visual reads. Individuals with SUVR ≥ 1.24 were considered amyloid positive, and those with SUVR < 1.10 were considered amyloid negative. Individuals with SUVR between 1.10 and 1.23 were considered amyloid‐positive if they also had a positive rating from at least two out of four trained raters. More details were previously specified.^32^

### 2.3 Plasma biomarkers

Ethylenediaminetetraacetic acid (EDTA) plasma was sampled, aliquoted, and frozen at −80°C. Plasma samples were analyzed at the Neurochemistry Laboratory, Amsterdam UMC, VUmc, the Netherlands, using a single molecule array (Simoa) HDx analyzer (Quanterix, Billerica, MA, USA). Aβ40 and Aβ42 were measured with the Simoa Neuro 4‐Plex E Kit, while p‐tau217 was measured using the Simoa p‐Tau‐217 CARe Advantage Kit. Samples were measured in singlicates for the Neuro 4‐Plex E Kit (concentration coefficient of variation [CV] < 5%) and in duplicates for the p‐tau217 kit. Two quality control samples were run on each plate for each analyte, except for p‐tau217, which had three control samples. Participants’ data were excluded from the analyses when the CV exceeded 20% and/or if the concentration was not between the assays’ lower and upper limits of quantification (*n* = 1).

### 2.4 Covariates

Based on the literature, we identified the most relevant variables that could play a role in the relationship between physical activity and AD biomarkers.^33^ We selected age (years), sex (female/male), APOE4 carriership (yes/no), education (primary, lower, intermediate, higher), the time between physical activity and Aβ biomarker assessments (years), season (spring/summer/autumn/winter), and estimated glomerular filtration rate (eGFR; only in plasma biomarker models) as potential confounders and controlled for these variables in all analyses for model 1. In a second model, we additionally controlled for body mass index (BMI, kg/m^2^), hypertension (yes/no), diabetes (yes/no), and smoking (yes/no). Education and smoking were measured during home visits. BMI, eGFR, hypertension, and diabetes were measured at the research center. BMI was calculated using height and weight. eGFR was calculated based on serum creatinine. Hypertension was defined as having systolic blood pressure > 140 mmHg or diastolic blood pressure > 90 mmHg or using blood pressure‐lowering medication. Diabetes was defined as fasting serum glucose ≥7.0 mmol/L or non‐fasting serum glucose levels ≥11.0 mmol/L or using blood‐glucose‐lowering medication.

### 2.5 Statistical analyses

All analyses were performed using R (version 4.1.2). We used robust regression models to investigate the association between each physical activity measure (awake sedentary time, LPA, MVPA, total physical activity) and three AD biomarkers (SUVR, Aβ42/Aβ40, p‐tau217), adjusting for potential confounders. Robust regression (R *robustbase* package) was chosen due to its ability to handle heteroscedasticity and outliers effectively without relying on the assumption of normally distributed residuals.^38^ These features make it particularly suitable for analyses involving complex biological processes where such issues are common. All variables were scaled before entering regression models. We also reported false discovery rate (FDR) *p*‐values to balance power and false positive results due to multiple comparisons.

For aim 1, we examined the relationship between physical activity and plasma AD biomarkers (Aβ42/Aβ40 and p‐tau217) with robust linear regression analysis, controlling for age, sex, APOE4 carriership, education, time between physical activity and AD biomarker assessments, season, and eGFR (model 1). We additionally controlled for BMI, hypertension, diabetes, and smoking (model 2).

For aim 2, we first investigated the association between physical activity and SUVR with robust linear regression analysis, controlling for the same covariates (excluding eGFR). We used continuous SUVR values in our primary analysis, as they retain more information and offer greater statistical power than a binary PET outcome. Then, we examined whether physical activity was associated with *incident* amyloid PET positivity, i.e., the onset of early AD pathology, using logistic regression analysis. To ensure participants were amyloid‐negative at baseline, we restricted the analysis to individuals with p‐tau217 < 0.42 pg/mL, in line with the threshold proposed by Ashton et al. (2024).^11^ This exclusion allowed us to focus on physical activity as a potential preventive factor, reduce confounding from ongoing disease processes, and establish a clearer temporal sequence.

Previous studies have suggested that the association between physical activity and AD biomarkers may be influenced by sex and apolipoprotein E4 (APOE4).^39^, ^40^, ^41^, ^42^ Additionally, as risk factors can exert different effects across the lifespan, understanding potential age‐related modifications is crucial for identifying the most effective windows for prevention.^2^ To explore these potential modification effects, we performed interaction analyses to test whether age, sex, or APOE4 moderated the relationship between physical activity and AD biomarkers by separately adding product terms (physical activity*age/sex/APOE4) to the models while controlling for all covariates in the main analysis.

To address potential selection bias in the current sample, we performed sensitivity analyses for aims 1 and 2 using inverse probability weighting based on age, sex, and education.

For aim 3, we performed compositional isotemporal substitution analyses using the *Compositions* R package (v2.0‐8).^22^, ^34^ Specifically, we examined the association between theoretically replacing 30 minutes of other activities (sleep or awake sedentary time) with physical activity (LPA or MVPA) and levels of AD biomarkers.^34^ Isometric log‐ratio (ILR) coordinates for all pairwise substitutions were computed and entered into linear regression models as independent variables, controlling for age, sex, APOE4 carriership, education, time between physical activity and AD biomarker assessments, season, and eGFR (only for plasma biomarkers). Rather than comparing activities in isolation, ILR coordinates show the ratio of time spent in one activity relative to others, allowing us to assess how theoretical changes in activity distribution influence outcomes. Further details about this analysis were previously published.^34^

## 3 RESULTS

### 3.1 Sample characteristics

Table 1 presents the characteristics of the 242 included participants. The mean ages at accelerometry, plasma biomarkers, and PET imaging were 63.49 (5.61), 63.37 (5.64), and 70.14 (5.51) years, respectively. Approximately half of the sample was female (47.1%), and 37.6% of the participants had completed higher education. The mean time between accelerometry and plasma AD biomarkers was 0.15 years (SD = 0.42), and between accelerometry and PET imaging was 6.66 years (SD = 0.93).

**TABLE 1:** Sample characteristics.

Variables	Levels	Overall
N		242
Years between accelerometry and plasma biomarkers, mean (SD)		0.15 (0.42)
Years between accelerometry and PET scan, mean (SD)		6.66 (0.93)
**Demographic information**		
Age at accelerometry, mean (SD)		63.49 (5.61)
Age at plasma, mean (SD)		63.37 (5.64)
Age at PET, mean (SD)		70.14 (5.51)
Sex (%)	Female	114 (47.1)
	Male	128 (52.9)
Education (%)	Primary	17 (7.0)
	Lower	64 (26.4)
	Intermediate	70 (28.9)
	Higher	91 (37.6)
**Genetic measures**		
Number of APOE4 alleles (%)	0	172 (71.1)
	1	60 (24.8)
	2	10 (4.1)
**Biomarker measures**		
Amyloid PET status (%)	Negative	196 (81.0)
	Positive	46 (19.0)
SUVR, mean (SD)		1.05 (0.18)
Plasma Aβ42/Aβ40 ratio, mean (SD)		0.06 (0.01)
Plasma p‐tau217 (pg/mL), mean (SD)		0.30 (0.22)
**Accelerometry measures**		
Light physical activity (min/day), mean (SD)		152 (29)
Moderate‐vigorous physical activity (min/day), mean (SD)		94 (27)
Awake sedentary time (min/day), mean (SD)		794 (80)
Sleep time (min/day), mean (SD)		389 (65)
Total physical activity (min/day), mean (SD)		246.11 (52.86)
**Covariates at accelerometry**		
BMI (kg/m^2^), mean (SD)		27.42 (4.00)
Hypertension (%)	No	103 (42.6)
	Yes	139 (57.4)
Diabetes (%)	No	225 (93.4)
	Yes	16 (6.6)
Smoking (%)	No	202 (83.8)
	Yes	39 (16.2)
Depressive symptoms CESD scale, mean (SD)		4.91 (6.34)
eGFR (µmol/L), mean (SD)		84.25 (12.78)

Abbreviations: Aβ42/Aβ40, amyloid‐beta 42 to amyloid‐beta 40 ratio; APOE4, apolipoprotein E4; BMI, body mass index; CESD, Centre for Epidemiological Studies Depression Scale; eGFR, estimated glomerular filtration rate; PET, positron emission tomography; p‐tau217, phosphorylated tau at threonine 217; SD, standard deviation; SUVR, standard uptake value ratio.

### 3.2 Association between physical activity and plasma AD biomarkers

**FIGURE 2 alz70655-fig-0002:**
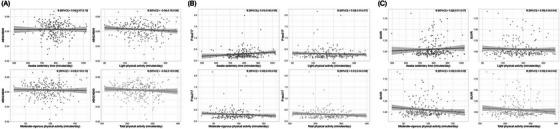
Scatterplot showing association between physical activity and AD biomarkers: (A) plasma Aβ42/Aβ40; (B) plasma p‐tau217; (C) Aβ PET SUVR. Aβ, beta‐amyloid; AD, Alzheimer's disease; PET, positron emission tomography; SUVR, standard uptake value ratio.

In cross‐sectional analysis, no statistically significant associations were observed between plasma Aβ42/Aβ40 and any physical activity measures (Table 2; Figure 2A). For example, a 1 SD decrease in awake sedentary time was associated with a 0.04 SD change in Aβ42/Aβ40, but the 95% confidence interval [−0.07 to 0.15] included zero, indicating no significant association (*p *= 0.421; model 1). Similarly, a 1 SD increase in MVPA was associated with a 0.03 SD change in Aβ42/Aβ40, but again the confidence interval was wide [−0.16 to 0.10], indicating no significant association (*p *= 0.602; model 1). All results were comparable for model 2, controlling additionally for vascular risk factors.

**TABLE 2:** Associations of physical activity and plasma AD biomarkers.

		Model 1			Model 2		
		β		*p*	β		*p*
AD biomarkers	Physical activity	(95% CI)	*p*	[FDR]	(95% CI)	*p*	[FDR]
Aβ42/Aβ40	Awake sedentary time (minutes/day)	0.04 (−0.07,0.15)	0.421	0.948	0.06 (−0.05,0.17)	0.306	0.840
	LPA (minutes/day)	−0.04 (−0.16,0.08)	0.448	0.948	−0.06 (‐0.18,0.06)	0.332	0.840
	MVPA (minutes/day)	−0.03 (−0.16,0.10)	0.602	0.948	−0.06 (‐0.21,0.09)	0.395	0.840
	Total physical activity (minutes/day)	−0.04 (−0.16,0.08)	0.495	0.948	−0.06 (‐0.19,0.07)	0.335	0.840
p‐tau217	Awake sedentary time (minutes/day)	0.01 (−0.06,0.08)	0.755	0.948	0.01 (−0.06,0.08)	0.823	0.955
	LPA (minutes/day)	0.02 (−0.03,0.07)	0.501	0.948	0.02 (−0.03,0.07)	0.391	0.840
	MVPA (minutes/day)	0.00 (−0.05,0.05)	0.871	0.948	0.01 (−0.05,0.07)	0.686	0.947
	Total physical activity (minutes/day)	0.01 (−0.04,0.06)	0.642	0.948	0.02 (−0.04,0.08)	0.490	0.840

Abbreviations: AD, Alzheimer's disease; APOE4, apolipoprotein E4; CI, confidence interval; FDR, false discovery rate; LPA, light physical activity; MVPA, moderate–vigorous physical activity; β, standardized beta coefficient.

*Note*: Model 1: age at plasma + APOE4 carriership + sex + education + time between physical activity and plasma biomarkers + season + eGFR (linear robust regression). Model 2: Model 1 + BMI + hypertension + diabetes + smoking (linear robust regression).

Next, we investigated the cross‐sectional association between the different physical activity measures and plasma p‐tau217 as an outcome (Table 2; Figure 2B). All associations were small and not significant. For example, the regression analysis of MVPA on p‐tau217 yielded a β = 0.00, 95% confidence interval of [−0.05 to 0.05], and thus an insignificant association (*p *= 0.871).

Sensitivity analyses using inverse probability weighting produced similar findings (Table S2).

### 3.3 Association between physical activity and Aβ PET

We investigated the association between physical activity at baseline and amyloid PET burden (SUVR) at an average follow‐up time of 7 years. No significant association was observed (Table 3, Figure 2C). For example, awake sedentary time corresponded with a 0.03 [−0.01 to 0.07] SD increase in SUVR, though it was not statistically significant (*p *= 0.198). No association was observed between MVPA and SUVR (β = 0.00 [−0.05, 0.05], *p *= 0.901, model 1). Similar findings were seen for LPA (*p *= 0.805) and total physical activity (*p *= 0.948). In sensitivity analyses using inverse probability weighting, our results remained virtually the same (Table S3).

**TABLE 3 alz70655-tbl-0003:** Associations of physical activity and SUVR Aβ PET.

	Model 1			Model 2		
	β	*p*‐value	*p*‐value	β	*p*‐value	*p*‐value
Physical activity	(95% CI)	*p*‐value	[FDR]	(95% CI)	*p*‐value	[FDR]
Awake sedentary time (minutes/day)	0.03 (−0.01,0.07)	0.198	0.948	0.02 (−0.03,0.07)	0.430	0.840
LPA (minutes/day)	0.00 (−0.04,0.04)	0.805	0.948	0.00 (−0.05,0.05)	0.957	0.957
MVPA (minutes/day)	0.00 (−0.05,0.05)	0.901	0.948	0.01 (−0.04,0.06)	0.710	0.947
Total physical activity (minutes/day)	0.00 (−0.04,0.04)	0.948	0.948	0.00 (−0.05,0.05)	0.875	0.955

Abbreviations: Aβ, beta‐amyloid; APOE4, apolipoprotein E4; β, standardized beta coefficient; CI, confidence interval; FDR, false discovery rate; LPA, light physical activity; MVPA, moderate‐vigorous physical activity; PET, positron emission tomography; SUVR, standardized uptake value ratio.

Model 1: age at PET + APOE4 carriership + sex + education + time between physical activity and PET + season (linear robust regression).

Model 2: Model 1 + BMI + hypertension + diabetes + smoking (linear robust regression).

As an alternative to SUVR, we examined whether physical activity was associated to *incident* Aβ PET positivity as an outcome. We only included participants with low p‐tau217 levels at baseline (p‐tau217 < 0.42pg/mL^11^; *n* = 205) to exclude those with ongoing pathology at the time physical activity was measured, and to assess the association of physical activity with the onset of AD pathology. Again, no associations were found, e.g., for awake sedentary time (OR = 1.11 [0.70, 1.80], *p *= 0.651) or MVPA (OR = 0.99 [0.62, 1.57], *p *= 0.965; Table S4).

### 3.4 Moderation effects of age, sex, and APOE4

We examined whether age, sex, or APOE4 carriership moderated the relationship between physical activity and AD biomarkers. Effect sizes for interaction[Fig alz70655-fig-0002], [Table alz70655-tbl-0003] were small, with β estimates generally close to zero. None of the three potential modifiers showed a significant influence (all p‐for‐interaction > 0.05). Full results are presented in Tables S5 and S6.

### 3.5 Association between reallocation of daily activities and AD biomarkers

**FIGURE 3:**
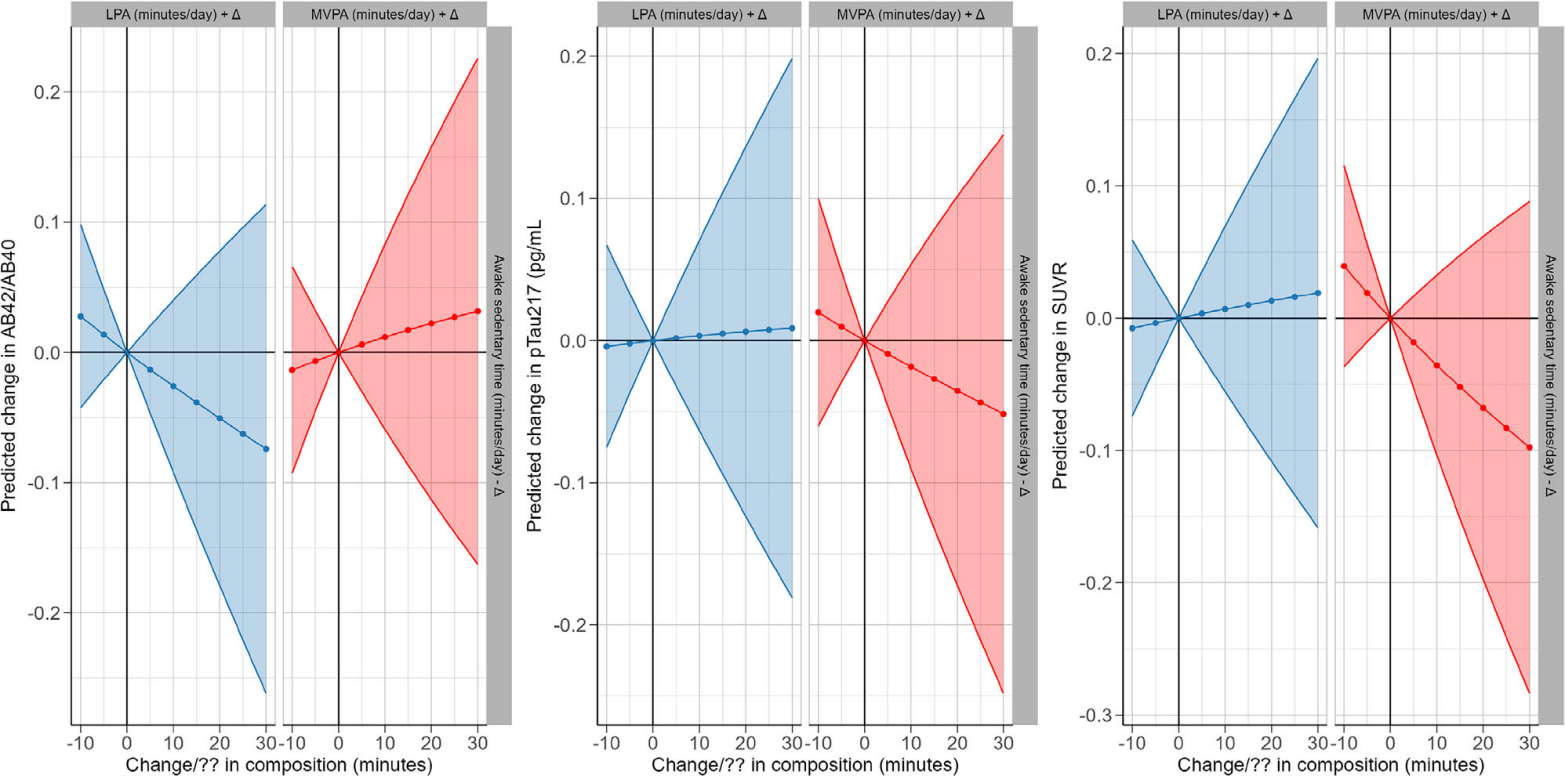
Estimated changes in AD biomarkers following reallocation of awake sedentary time to physical activity, Values represent the estimated changes in AD biomarker levels compared to the mean time‐use composition of the study population, under a hypothetical scenario where 30 minutesof awake sedentary time are reallocated to either light physical activity or moderate‐to‐vigorous physical activity, while other behaviors remain constant. For example, a 30‐min reallocation to moderate‐to‐vigorous physical activity was associated with an estimated 0.1 SD reduction in p‐tau217 levels. Model was adjusted for age, sex, APOE4 cerriership, education, time between physical activity and outcome, season, and eGFR (only plasma biomarkers).

In the compositional data analyses, theoretical reallocation of 30 minutes spent on awake sedentary time with 30 minutes spent on physical activity (either LPA or MVPA) was not significantly associated with any AD biomarkers (Table 4). For the Aβ42/Aβ40 ratio, the estimated changes were minimal (LPA: β = −0.02 [−0.09, 0.04]; MVPA: β = 0.01 [−0.06, 0.08]). For p‐tau217, reallocation of 30 minutes to MVPA was associated with a non‐significant decrease of 0.1 SD [‐0.28, 0.09], while reallocation to LPA showed a small, non‐significant increase of 0.02 SD [−0.16, 0.20]. Similarly, for SUVR, the estimated changes were β = 0.02 [−0.16, 0.20] (LPA) and β = −0.10 [−0.28, 0.09] (MVPA). Figure 3 illustrates these estimated changes across biomarkers.

**TABLE 4:** Association between 30‐minute activity reallocation and AD biomarkers.

Parameter		With 30 minutes of							
		Sleep time		Awake sedentary time		LPA		MVPA	
		β	95% CI	β	95% CI	β	95% CI	β	95% CI
**Aβ42/Aβ40**									
Replacing 30 minutes of	Sleep time (minutes/day)			0.00	−0.02 to 0.02	−0.02	−0.09 to 0.05	0.01	−0.06 to 0.08
	Awake sedentary time (minutes/day)	0.00	−0.02 to 0.02			−0.02	−0.09 to 0.04	0.01	−0.06 to 0.08
	LPA (minutes/day)	0.02	−0.05 to 0.10	0.03	−0.04 to 0.10			0.03	−0.10 to 0.17
	MVPA (minutes/day)	−0.01	−0.09 to 0.07	−0.01	−0.09 to 0.07	−0.03	−0.17 to 0.10		
**P‐tau217 (pg/mL)**									
Replacing 30 minutes of	Sleep time (minutes/day)			0.00	−0.05 to 0.06	0.02	−0.16 to 0.21	−0.09	−0.28 to 0.10
	Awake sedentary time (minutes/day)	−0.01	−0.06 to 0.05			0.02	−0.16 to 0.20	−0.10	−0.28 to 0.09
	LPA (minutes/day)	−0.03	−0.25 to 0.19	−0.02	−0.24 to 0.19			−0.12	−0.50 to 0.26
	MVPA (minutes/day)	0.13	−0.13 to 0.39	0.13	−0.13 to 0.39	0.15	−0.26 to 0.57		
**SUVR**									
Replacing 30 minutes of	Sleep time (minutes/day)			0.00	−0.05 to 0.06	0.02	−0.16 to 0.21	−0.09	−0.28 to 0.10
	Awake sedentary time (minutes/day)	0.00	−0.01 to 0.01			0.02	−0.16 to 0.20	−0.10	−0.28 to 0.09
	LPA (minutes/day)	−0.01	−0.05 to 0.04	−0.02	−0.24 to 0.19			−0.12	−0.50 to 0.26
	MVPA (minutes/day)	0.02	−0.02 to 0.07	0.13	−0.13 to 0.39	0.15	−0.26 to 0.57		

Values represent the estimated differences in biomarker levels between the mean composition of the study sample and a new composition, where, for example, 30 minutes more are spent on moderate‐to‐vigorous physical activity and 30 minutes less in sedentary time (while light physical activity and sleep remain unchanged). Model was adjusted for age, sex, APOE4 carriership, education, time between physical activity and outcome, season, and eGFR (only plasma biomarkers).

Abbreviations: Aβ42/Aβ40, amyloid‐beta 42 to amyloid‐beta 40; AD, Alzheimer's disease; APOE4, apolipoprotein E4; β, standardized beta coefficient; CI, confidence interval; LPA, light physical activity; MVPA, moderate‐vigorous physical activity; p‐tau217, phosphorylated tau at threonine 217; SUVR, standard uptake value ratio.

## 4 DISCUSSION

Previous studies investigating the link between physical activity and AD biomarkers mostly relied on self‐reported activity data and have produced mixed results. Our study aimed to examine this relationship in greater depth. Physical activity was assessed using accelerometry at baseline, plasma Aβ42/Aβ40 and plasma p‐tau217 were measured within 1 year of physical activity, and PET‐derived Aβ was collected 7 years later. We found: (1) no association between physical activity and plasma Aβ42/Aβ40 or plasma p‐tau217 cross‐sectionally; (2) physical activity was not associated with PET‐derived SUVR Aβ 7 years later; (3) age, sex, and APOE4 did not modify the relationship between physical activity and AD biomarkers; and (4) accounting for the intercorrelation among activities through compositional analysis did not alter the results.

Our analysis revealed no association between physical activity and plasma Aβ42/Aβ40 or plasma p‐tau217. Previously, observational studies employing self‐reported physical activity data have shown some support for the link between higher physical activity levels and lower Aβ42/Aβ40.^17^, ^43^ A longitudinal study utilizing the Pittsburgh Cardiovascular Health Study Cognition Study data (*n* = 149) revealed that self‐reported physical activity at baseline was predictive of lower Aβ42/Aβ40 9–13 years later.^43^ Conversely, a 6‐month exercise intervention found no change in the Aβ42/Aβ40 ratio between pre‐ versus post‐intervention.^14^ This aligns well with a meta‐analysis by Rodriguez‐Ayllon et al. (2023), showing no association between physical activity and plasma Aβ.^15^ To date, only one study has examined the link between self‐reported physical activity and plasma p‐tau217, reporting a negative association, but only in the highest quartile of physical activity.^18^ This study included participants with cognitive impairment and AD dementia, which may limit the generalizability of the findings to the preclinical phase. One potential explanation is that, similar to the gradual accumulation of AD pathology, the effect of physical activity is subtle and requires an extended period before becoming detectable. The habit of doing physical activity can vary significantly over time, adding another layer of complexity to the relationship. This highlights a critical gap in the literature, as no longitudinal studies have explored the association between objectively measured physical activity and AD pathology over time.

Using PET‐derived SUVR seven years later, we confirmed that physical activity was not associated with brain Aβ. This finding aligns with a previous study in the RS, which found no association between self‐reported physical activity and Aβ PET burden.^32^ Only three previous studies had assessed physical activity objectively, and our result is consistent with theirs.^19^, ^20^, ^21^ Marino et al. (2024) found no differences in overall daily physical activity between Aβ‐positive and ‐negative individuals using accelerometry.^20^ In time‐of‐day analyses, Aβ‐positive individuals showed lower activity time and variability of overnight/early evening physical activity. It is possible that patterns of physical activity based on the time of day, rather than the total amount or intensity of daily physical activity, may be related to AD pathology. Supporting this notion, our previous work found greater intradaily variability, reflecting a more fragmented 24‐h activity rhythm, was associated with increased brain Aβ.^44^ Several other studies have also linked activity fragmentation to cognitive decline.^45^, ^46^ These findings emphasize the importance of examining activity patterns in future research on AD biomarkers. The type and context of physical activity might also play a role.^47^ Activities that also incorporate cognitive or social engagement may register as lower in quantity but offer distinct neuroprotective benefits.^48^, ^49^, ^50^ Future studies should aim to account for this complexity by integrating measures that reflect the diverse dimensions and contexts of physical activity.

We investigated whether the associations between physical activity and AD biomarkers differ by age, sex, or APOE4 status, given plausible biological and behavioral differences across these groups.^2^ APOE4 is the strongest genetic risk factor for AD and may influence both AD pathology as well as the brain's response to lifestyle interventions. Hormonal changes in females throughout the lifespan could interact with physical activity's effects on AD biomarkers accumulation.^51^ The neurobiological benefits of physical activity may vary across the life course due to age‐related changes in neuroplasticity and vascular health.^52^ Our study found no moderation effect of age, sex, or APOE4, which aligns with the current state of research, particularly for APOE4.^33^, ^41^, ^42^, ^53^ In a longitudinal study using data from the Australian Imaging, Biomarkers and Lifestyle Study of Ageing (*n* = 731), APOE4 carriership did not moderate the relationship between self‐reported physical activity and brain Aβ over time.^53^ The roles of age and sex have been less thoroughly investigated. Two previous studies identified an association between physical activity and Aβ exclusively in men.^39^, ^40^ However, these studies relied on self‐reported questionnaires, and the participants were older (averaging 66.4–69.33 years) at the time of data collection. These findings underscore the need for future research to explore the role of age and sex in more diverse populations, utilizing objective measures of physical activity.

In compositional isotemporal substitution analyses, we found no significant association between reallocating 30 minutes of other activities to physical activity and AD biomarkers. Some estimated effects—such as SUVR reduction associated with reallocating time to MVPA—approached the clinically meaningful threshold in our cohort (0.08 SUVR, the average increase in SUVR observed over 10 years).^32^ The wide confidence intervals and lack of statistical significance indicate these results are not robust and should be interpreted cautiously. Previous studies mainly employed compositional analysis to study physical activity and cognition or cardiometabolic health.^23^, ^24^, ^25^, ^26^ To our knowledge, this is one of the first studies using compositional isotemporal substitution analyses to examine the relationship between physical activity and AD biomarkers.^54^ Our findings do not reflect that replacing 30 minutes of awake sedentary time with physical activity is not beneficial, but it may manifest through other mechanisms. A previous study using the RS data by Hofman et al. (2020) has shown that replacing sleep or awake sedentary time with MVPA was associated with fewer depressive symptoms.^34^ Given the established link between depressive symptoms and dementia, physical activity may benefit dementia risk through mental health, which could also serve as an early indicator.^55^, ^56^ Replacing awake sedentary time with physical activity is linked to a more favorable cardiometabolic profile, which may improve cerebrovascular functions and reduce AD pathology accumulation over time.^26^

Physical activity is associated with better cognitive function during aging and reduced risk of AD.^4^, ^57^ These associations are mediated by multiple pathways, such as changes in brain structure and function.^58^, ^59^ Specifically, many studies have shown that physical activity benefits several cognitive domains, most notably global cognition and executive function.^57^, ^58^, ^59^ These cognitive benefits operate through distinct but interconnected mechanisms involving cellular and molecular processes (e.g., growth factors), structural and functional brain adaptations (e.g., brain volume), and behavioral changes (e.g., sleep), with brain health serving as a key mediator.^59^, ^60^ In addition, cross‐sectional evidence shows that greater physical activity is associated with larger brain volumes (total brain volume, grey matter volume, white matter volume, hippocampal volume), reduced white matter hyperintensities, and increased cerebral blood flow.^61^, ^62^, ^63^, ^64^ However, findings from large‐scale bidirectional longitudinal studies, including the UK Biobank (*n* = 3,027, mean age = 62.45) and the Rotterdam Study (*n* = 4,365, mean age = 64.01), reveal more complex temporal dynamics.^65^, ^66^, ^67^ Although the association of physical activity with brain outcomes appears inconsistent, baseline brain health seems to predict consistently future physical activity levels, suggesting bidirectional relationships. Given the complex bidirectional relationships and variable findings, future research should further disentangle the directionality of these associations and investigate how they relate to AD biomarkers.^59^

While the current study included a substantial sample, a small association may have gone undetected. Larger studies are needed to further evaluate this relationship. This study has several limitations. First, the cross‐sectional design prevents causal inferences and limits investigation of dynamic AD biomarker changes. Longitudinal studies with repeated measurements are needed to establish any temporal relationship. Second, the accelerometry data were calibrated to a generic acceleration threshold, assuming a uniform metabolic demand to perform an activity across populations, which may not be valid.^68^, ^69^ Future research should employ population‐standardized values. Third, our study focuses on late midlife and does not account for earlier life periods, though physical activity earlier in life may influence later AD biomarker accumulation.^6^, ^10^, ^13^ Future research should evaluate physical activity across the lifespan. Lastly, our analysis drew from a sub‐cohort of the population‐based Rotterdam Study, which may reflect a selection bias toward a relatively homogenous sample—younger, white, highly educated, male, and physically active. Physical activity is a complex behavior that is often influenced by educational, social, and cultural background and varies by age, sex, and other demographic factors. Future studies should aim to include more diverse populations and objective measures with refined activity thresholds to validate and expand upon these findings.

## 5 CONCLUSION

This study found no association between physical activity and AD burden. Age, sex, and APOE4 did not modify this relationship. In a hypothetical scenario, reallocating 30 minutes of awake sedentary time to physical activity resulted in no difference in AD biomarkers.

## CONFLICT OF INTEREST STATEMENT

The authors report no conflict of interest.

## CONSENT STATEMENT

The Rotterdam Study has been approved by the Medical Ethics Committee of the Erasmus MC (MEC‐2018‐085) and by the Dutch Ministry of Health, Welfare, and Sport (Population Screening Act WBO, license number 1071272‐159521‐PG). Written informed consent was obtained from all participants.

## DIVERSITY, EQUITY, AND INCLUSION STATEMENT

We are committed to promoting diversity, equity, and inclusion in all aspects of our research. This study was designed and conducted with consideration for equitable representation of participants and rigorous methodology to reduce potential biases. We acknowledge ongoing disparities in health research participation and data availability across racial, ethnics, socioeconomic, and other demographic groups. While our sample may not fully reflect the diversity of the broader population, we recognize the importance of inclusive research practices and are committed to improving representation in future studies through inclusive recruitment strategies and community engagement.

## Supporting information



Supporting Information

Supporting Information
