# Supplementary material for: Objective physical activity and Alzheimer's disease burden in the population‐based Rotterdam Study
Source: Alzheimers Dement. 2025 Sep 18;21(9):e70655. doi: 10.1002/alz.70655 (PMC12445197; doi:10.1002/alz.70655)
Supplement: Supplementary file 1 — Supporting Information [file ALZ-21-e70655-s001.docx]

**Table S1.** Sample characteristics of participants who were eligible for PET imaging versus participants included in this study

| **Variables** | **Levels** | **Eligible for PET imaging** | **Included in the analysis** | **p** |
| --- | --- | --- | --- | --- |
| n |  | 1826 | 242 |  |
| Age, mean (SD) |  | 70.76 (6.66) | 68.51 (5.03) | <0.001 |
| Sex (%) | Male | 782 (42.8) | 128 (52.9) | 0.004 |
|  | Female | 1044 (57.2) | 114 (47.1) |  |
| Education (%) | Primary | 121 (6.7) | 17 (7.0) | 0.001 |
|  | Lower | 664 (36.7) | 64 (26.4) |  |
|  | Intermediate | 547 (30.2) | 70 (28.9) |  |
|  | Higher | 479 (26.4) | 91 (37.6) |  |
| APOE e4 carriership (%) | No | 1086 (70.8) | 172 (71.1) | 0.990 |
|  | Yes | 448 (29.2) | 70 (28.9) |  |
| BMI, mean (SD) |  | 27.30 (4.06) | 27.43 (3.99) | 0.643 |
| Diabetes (%) | No | 1604 (89.1) | 215 (90.3) | 0.820 |
|  | Yes | 196 (10.9) | 23 (9.7) |  |
| Hypertension (%) | No | 660 (36.4) | 103 (42.6) | 0.075 |
|  | Yes | 1151 (63.6) | 139 (57.4) |  |
| Smoking (%) | No | 1524 (84.5) | 202 (83.8) | 0.864 |
|  | Yes | 280 (15.5) | 39 (16.2) |  |
| Depressive symptoms, mean (SD) |  | 5.36 (7.24) | 4.91 (6.34) | 0.354 |

**Table S2.** Associations of physical activity and plasma AD biomarkers (with inverse probability weighting)

| **Aβ biomarkers** | **Physical activity** | **Model 1** | | | **Model 2** | | |
| --- | --- | --- | --- | --- | --- | --- | --- |
|  |  | **β** | **p** | **p** | **β** | **p** | **p** |
|  |  | **(95% CI)** |  | **[FDR]** | **(95% CI)** |  | **[FDR]** |
| Aβ42/Aβ40 | Awake sedentary time (minutes/day) | 0.06 (-0.04,0.16) | 0.222 | 0.883 | 0.08 (-0.02,0.18) | 0.121 | 0.624 |
|  | LPA (minutes/day) | -0.06 (-0.17,0.05) | 0.299 | 0.883 | -0.08 (-0.19,0.03) | 0.157 | 0.624 |
|  | MVPA (minutes/day) | -0.04 (-0.15,0.07) | 0.515 | 0.883 | -0.08 (-0.21,0.05) | 0.231 | 0.624 |
|  | Total physical activity (minutes/day) | -0.05 (-0.16,0.06) | 0.363 | 0.883 | -0.08 (-0.20,0.04) | 0.160 | 0.624 |
| P-tau217 (pg/mL) | Awake sedentary time (minutes/day) | 0.00 (-0.07,0.07) | 0.910 | 0.981 | -0.01 (-0.09,0.07) | 0.849 | 0.869 |
|  | LPA (minutes/day) | 0.03 (-0.03,0.09) | 0.406 | 0.883 | 0.03 (-0.03,0.09) | 0.276 | 0.624 |
|  | MVPA (minutes/day) | 0.01 (-0.05,0.07) | 0.653 | 0.980 | 0.02 (-0.04,0.08) | 0.464 | 0.696 |
|  | Total physical activity (minutes/day) | 0.02 (-0.04,0.08) | 0.483 | 0.883 | 0.03 (-0.03,0.09) | 0.336 | 0.624 |

Abbreviations: β, standardized beta coefficient; CI, confidence interval; FDR, false discovery rate; LPA; light physical activity; MVPA, moderate-vigorous physical activity.

Model 1: age at plasma + APOE4 carriership + sex + education + time between physical activity and plasma biomarkers + season + eGFR (linear robust regression)

Model 2: Model 1 + BMI + hypertension + diabetes + smoking (linear robust regression)

**Table S3.** Associations of physical activity and SUVR Aβ PET (with inverse probability weighting)

| **Physical activity** | **Model 1** | | | **Model 2** | | |
| --- | --- | --- | --- | --- | --- | --- |
|  | **β** | **p** | **p** | **β** | **p** | **p** |
|  | **(95% CI)** |  | **[FDR]** | **(95% CI)** |  | **[FDR]** |
| Awake sedentary time (minutes/day) | 0.02 (-0.02,0.06) | 0.287 | 0.883 | 0.02 (-0.02,0.06) | 0.364 | 0.624 |
| LPA (minutes/day) | 0.00 (-0.04,0.04) | 0.981 | 0.981 | 0.00 (-0.05,0.05) | 0.869 | 0.869 |
| MVPA (minutes/day) | 0.01 (-0.04,0.06) | 0.776 | 0.981 | 0.02 (-0.03,0.07) | 0.538 | 0.717 |
| Total physical activity (minutes/day) | 0.00 (-0.04,0.04) | 0.886 | 0.981 | 0.01 (-0.04,0.06) | 0.684 | 0.821 |

Abbreviations: β, standardized beta coefficient; CI, confidence interval; FDR, false discovery rate; LPA; light physical activity; MVPA, moderate-vigorous physical activity.

Model 1: age at PET + APOE4 carriership + sex + education + time between physical activity and PET + season (linear robust regression)

Model 2: Model 1 + BMI + hypertension + diabetes + smoking (linear robust regression)

**Table S4**. Associations of physical activity with Aβ PET positivity

| **Variable** | **Model 1** | | | **Model 2** | | |
| --- | --- | --- | --- | --- | --- | --- |
|  | **OR** | **p** | **p** | **OR** | **p** | **p** |
|  | **(95% CI)** |  | **[FDR]** | **(95% CI)** |  | **[FDR]** |
| Awake sedentary time (minutes/day) | 1.11 (0.70,1.80) | 0.651 | 0.965 | 1.20 (0.73,2.04) | 0.483 | 0.785 |
| LPA (minutes/day) | 0.97 (0.61,1.58) | 0.912 | 0.965 | 0.93 (0.55,1.59) | 0.785 | 0.785 |
| MVPA (minutes/day) | 0.99 (0.62,1.57) | 0.965 | 0.965 | 0.92 (0.52,1.57) | 0.756 | 0.785 |
| Total physical activity (minutes/day) | 0.98 (0.61,1.58) | 0.934 | 0.965 | 0.92 (0.53,1.58) | 0.755 | 0.785 |

Abbreviations: OR, odds ratio; CI, confidence interval; FDR, false discovery rate; LPA, light physical activity; MVPA, moderate-vigorous physical activity.

Reference=amyloid negative

Model 1: age at PET + APOE4 carriership + sex + education + time between physical activity and PET + season (logistic regression)

Model 2: Model 1 + BMI + hypertension + diabetes + smoking (logistic regression)

**Table S5**. Moderation effects of age, sex, APOE4 and physical activity on plasma AD biomarkers

|  | **Model 1** | | | **Model 2** | | |
| --- | --- | --- | --- | --- | --- | --- |
|  | **β** | **p** | **p** | **β** | **p** | **p** |
|  | **(95% CI)** |  | **[FDR]** | **(95% CI)** |  | **[FDR]** |
| **Aβ42/Aβ40** | | | | | | |
| Awake sedentary time * Age | -0.04 (-0.40,0.32) | 0.852 | 0.932 | -0.11 (-0.48,0.26) | 0.559 | 0.976 |
| Awake sedentary time * Sex | -0.04 (-0.39,0.31) | 0.845 | 0.940 | -0.10 (-0.44,0.24) | 0.569 | 0.980 |
| Awake sedentary time * APOE4 | -0.02 (-0.38,0.34) | 0.913 | 0.913 | -0.08 (-0.44,0.28) | 0.647 | 0.972 |
| LPA * Age | -0.02 (-0.40,0.36) | 0.932 | 0.932 | -0.08 (-0.46,0.30) | 0.684 | 0.976 |
| LPA * Sex | -0.02 (-0.39,0.35) | 0.935 | 0.940 | -0.08 (-0.45,0.29) | 0.689 | 0.980 |
| LPA * APOE4 | -0.03 (-0.41,0.35) | 0.877 | 0.913 | -0.09 (-0.46,0.28) | 0.648 | 0.972 |
| MVPA * Age | -0.05 (-0.44,0.34) | 0.804 | 0.932 | -0.12 (-0.52,0.28) | 0.567 | 0.976 |
| MVPA * Sex | -0.02 (-0.39,0.35) | 0.920 | 0.940 | -0.09 (-0.46,0.28) | 0.653 | 0.980 |
| MVPA * APOE4 | -0.03 (-0.40,0.34) | 0.874 | 0.913 | -0.10 (-0.47,0.27) | 0.615 | 0.972 |
| Total physical activity * Age | -0.03 (-0.42,0.36) | 0.869 | 0.932 | -0.10 (-0.49,0.29) | 0.622 | 0.976 |
| Total physical activity * Sex | -0.01 (-0.38,0.36) | 0.940 | 0.940 | -0.08 (-0.45,0.29) | 0.679 | 0.980 |
| Total physical activity * APOE4 | -0.03 (-0.41,0.35) | 0.876 | 0.913 | -0.09 (-0.46,0.28) | 0.632 | 0.972 |
| **P-tau217 (pg/mL)** | | | | | | |
| Awake sedentary time * Age | -0.24 (-0.54,0.06) | 0.114 | 0.579 | -0.23 (-0.53,0.07) | 0.137 | 0.663 |
| Awake sedentary time * Sex | -0.23 (-0.53,0.07) | 0.141 | 0.519 | -0.21 (-0.51,0.09) | 0.173 | 0.609 |
| Awake sedentary time * APOE4 | -0.20 (-0.51,0.11) | 0.200 | 0.600 | -0.19 (-0.49,0.11) | 0.215 | 0.654 |
| LPA * Age | -0.21 (-0.53,0.11) | 0.193 | 0.579 | -0.20 (-0.52,0.12) | 0.219 | 0.663 |
| LPA * Sex | -0.22 (-0.54,0.10) | 0.172 | 0.519 | -0.20 (-0.52,0.12) | 0.203 | 0.609 |
| LPA * APOE4 | -0.22 (-0.54,0.10) | 0.190 | 0.600 | -0.20 (-0.52,0.12) | 0.214 | 0.654 |
| MVPA * Age | -0.21 (-0.51,0.09) | 0.175 | 0.579 | -0.19 (-0.49,0.11) | 0.205 | 0.663 |
| MVPA * Sex | -0.22 (-0.54,0.10) | 0.173 | 0.519 | -0.20 (-0.51,0.11) | 0.199 | 0.609 |
| MVPA * APOE4 | -0.22 (-0.54,0.10) | 0.188 | 0.600 | -0.20 (-0.52,0.12) | 0.215 | 0.654 |
| Total physical activity * Age | -0.21 (-0.52,0.10) | 0.188 | 0.579 | -0.20 (-0.51,0.11) | 0.221 | 0.663 |
| Total physical activity * Sex | -0.22 (-0.54,0.10) | 0.172 | 0.519 | -0.20 (-0.51,0.11) | 0.201 | 0.609 |
| Total physical activity * APOE4 | -0.22 (-0.54,0.10) | 0.193 | 0.600 | -0.20 (-0.52,0.12) | 0.218 | 0.654 |

Abbreviations: β, standardized beta coefficient; CI, confidence interval; FDR, false discovery rate; LPA, light physical activity; MVPA, moderate-vigorous physical activity; Aβ42/Aβ40, amyloid-beta 42 to amyloid-beta 40; p-tau217, phosphorylated tau at threonine 217; APOE4, Apolipoprotein E4.

Model 1: age at plasma + APOE4 carriership + sex + education + time between physical activity and PET+ season + eGFR (linear robust regression)

Model 2: Model 1 + BMI + hypertension + diabetes + smoking (linear robust regression)

**Table S6**. Moderation effects of with age, sex, APOE4 and physical activity on SUVR Aβ PET

|  | **Model 1** | | | **Model 2** | | |
| --- | --- | --- | --- | --- | --- | --- |
|  | **β** | **p** | **p** | **β** | **p** | **p** |
|  | **(95% CI)** |  | **[FDR]** | **(95% CI)** |  | **[FDR]** |
| **Awake sedentary time (minutes/day)** | | | | | | |
| Age interaction | -0.02 (-0.29,0.25) | 0.890 | 0.932 | 0.01 (-0.25,0.27) | 0.923 | 0.976 |
| Sex interaction | -0.05 (-0.31,0.21) | 0.686 | 0.940 | -0.02 (-0.26,0.22) | 0.857 | 0.980 |
| APOE4 interaction | -0.02 (-0.28,0.24) | 0.906 | 0.913 | 0.01 (-0.23,0.26) | 0.954 | 0.992 |
| **LPA (minutes/day)** | | | | | | |
| Age interaction | -0.02 (-0.29,0.25) | 0.898 | 0.932 | 0.00 (-0.26,0.26) | 0.976 | 0.976 |
| Sex interaction | -0.03 (-0.28,0.22) | 0.844 | 0.940 | 0.00 (-0.24,0.24) | 0.980 | 0.980 |
| APOE4 interaction | -0.03 (-0.30,0.24) | 0.841 | 0.913 | 0.00 (-0.25,0.25) | 0.972 | 0.992 |
| **MVPA (minutes/day)** | | | | | | |
| Age interaction | -0.02 (-0.29,0.25) | 0.877 | 0.932 | -0.01 (-0.27,0.25) | 0.956 | 0.976 |
| Sex interaction | -0.05 (-0.30,0.20) | 0.690 | 0.940 | -0.02 (-0.26,0.22) | 0.846 | 0.980 |
| APOE4 interaction | -0.03 (-0.30,0.24) | 0.831 | 0.913 | 0.00 (-0.25,0.25) | 0.985 | 0.992 |
| **Total physical activity (minutes/day)** | | | | | | |
| Age interaction | -0.02 (-0.29,0.25) | 0.901 | 0.932 | 0.00 (-0.26,0.26) | 0.970 | 0.976 |
| Sex interaction | -0.04 (-0.29,0.21) | 0.781 | 0.940 | -0.01 (-0.25,0.23) | 0.931 | 0.980 |
| APOE4 interaction | -0.03 (-0.29,0.23) | 0.841 | 0.913 | 0.00 (-0.25,0.25) | 0.992 | 0.992 |

Abbreviations: β, standardized beta coefficient; CI, confidence interval; FDR, false discovery rate; LPA, light physical activity; MVPA, moderate-vigorous physical activity; APOE4, Apolipoprotein E4.

Model 1: age at PET + APOE4 carriership + sex + education + time between physical activity and PET + season (logistic regression)

Model 2: Model 1 + BMI + hypertension + diabetes + smoking (logistic regression)
